# Role of vitamin D as adjuvant therapy on multiple sclerosis: an updated systematic review and meta-analysis of randomized controlled trials

**DOI:** 10.1186/s40001-025-02981-x

**Published:** 2025-08-12

**Authors:** Ibrahim Serag, Mohamed Abouzid, Khalid Radwan Alsaadany, Mohamed Hendawy, Hossam Tharwat Ali, Yazan Yaseen, Mostafa Hossam El din Moawad

**Affiliations:** 1 Faculty of Medicine, Mansoura University, Mansoura, Egypt; 2 Department of Physical Pharmacy and Pharmacokinetics, Faculty of Pharmacy, Poznan University of Medical Sciences, Rokietnicka 3 street, 60-806 Poznan, Poland; 3 Doctoral School, Poznan University of Medical Sciences, 60-812 Poznan, Poland; 4 Faculty of Pharmacy, Alexandria University, Alexandria, Egypt; 5 Qena Faculty of Medicine, South Valley University, Qena, Egypt; 6 Faculty of Medicine, Mutah University, Mutah, Jordan; 7 Alexandria Main University Hospital, Alexandria, Egypt; 8 Faculty of Medicine, Suez Canal University, Ismailia, Egypt

**Keywords:** Multiple sclerosis, Vitamin D, Cholecalciferol, Disease-modifying therapy

## Abstract

**Background:**

Multiple sclerosis (MS) is the most common demyelinating disorder affecting the central nervous system, with multiple risk factors suggested to be involved in the pathogenesis. Many studies have linked vitamin D deficiency to an increased risk of MS. This review aims to comprehensively evaluate the published randomized clinical trials (RCTs) of vitamin D supplements as add-on therapy for MS patients.

**Methods:**

We systematically searched the Web of Science, Scopus, PubMed, and Cochrane databases up to August 2024 for the published RCTs evaluating the use of vitamin D for MS in adults. All included studies were screened and abstracted independently by two authors. Radiological and clinical outcomes were extracted, and the meta-analysis was conducted using Review Manager 5.4.

**Results:**

Our meta-analysis, which included 21 studies with 1,903 patients (20.1% males), found that vitamin D supplementation significantly reduced expanded disability status scale scores (MD = − 0.17, p = 0.03), relapse rates (OR = 0.66, p = 0.02), and new T2 lesion formation (MD = − 0.48, p = 0.03) in patients with MS compared to controls, with minimal to no heterogeneity. However, there was no effect on the annual relapse rate (p = 0.81), timed 25-foot walk (p = 0.54), fatigue severity, or quality of life. Subgroup analysis indicated a relapse rate reduction only in those treated for more than 12 months (OR = 0.53, p = 0.003), suggesting duration-dependent benefits of vitamin D.

**Conclusions:**

Vitamin D supplementation produces statistically significant—yet clinically modest—reductions in disability progression, relapses, and new T2-lesion formation without demonstrable effects on fatigue or quality of life. Accordingly, it should be considered a potentially helpful adjunct pending more definitive evidence. Larger, dose-stratified trials powered for clinically meaningful endpoints are still needed before vitamin D can be endorsed as an efficacy-proven disease-modifying therapy.

## Introduction

Multiple sclerosis (MS) is the most common demyelinating disorder affecting the central nervous system [1]. With around 2.9 million patients living with MS, it is an increasing cause of disability worldwide, especially among young adults [2]. MS is thought to have an immune-mediated pathology that evolves into demyelination, axonal, and oligodendrocyte damage [3]. MS can be of distinct clinical subtypes, although relapsing–remitting MS affects around 80% of patients with a variety of symptoms, including vision, motor, and sensory symptoms, fatigue, incoordination, and cognitive impairment [4, 5]. Currently, disease-modifying therapies (DMT) target the underlying immune process to reduce relapses and lesion development and thus reduce possible disability over time [4–6]. Preclinical studies are conducted for potential gene therapies that can outweigh the benefits of DMT [6].

Although autoimmune pathology is the prevailing theory for MS, the specific etiology is not clear, and several environmental and other risk factors can trigger autoimmune attacks or interfere with the immune system's function and maturation [7]. A good understanding of and mitigation of preventable factors could be a basis for lessening the burden of the disease. Many studies have linked vitamin D deficiency, since the 1970 s, to increased risk of MS either directly or indirectly through sunlight exposure, latitude, or diet [8–11]. The observations are that high or proper vitamin D intake can prevent or reduce the risk of MS [8, 12–14]. In established MS patients, correlations between vitamin D levels and disease activity were concluded from some observational studies [8, 15, 16]. The first experimental study investigating vitamin D supplements for MS patients in 1986 showed that vitamin D, calcium, and magnesium supplements reduced relapse rates [17]. It is noteworthy that the following studies, up to date, show contradictory results regarding the therapeutic benefits of vitamin D supplements for patients with MS [17–21].

Notably, recent research has also identified emerging evidence linking vitamin D to outcomes in viral infections, particularly the severity of coronavirus disease 2019 (COVID-19) in MS patients. For instance, Montini et al. (2023) demonstrated that higher vitamin D levels may reduce the severity of COVID-19 in individuals with MS [22]. This finding adds urgency to understanding vitamin D's broader therapeutic potential in MS, especially in the context of viral infections, which may interact with autoimmune pathophysiology or affect disease management strategies [7].

Current guidelines do not recommend prescribing vitamin D solely for treating MS unless there is evidence of vitamin D deficiency [23]. Many reviews have attempted to study the effects of vitamin D on MS patients without the pooled therapeutic effects of vitamin D [1, 7]. The most recent systematic review and meta-analysis of only nine studies with 867 patients showed no benefits of high-dose cholecalciferol on expanded disability status scale (EDSS), annualized relapse rate (ARR), or new T2 lesions [24]. Notably, the included study exhibited several methodological limitations [1, 7, 24]. The previous reviews included limited studies from certain databases that assessed only two or three outcomes. Therefore, we conducted the present updated systematic review and meta-analysis to provide an updated comprehensive evaluation of the published randomized controlled trials (RCTs) addressing the benefits of vitamin D in different forms and dosages on a wide variety of primary and secondary outcomes. The review will also discuss the limitations and challenges of the available trials and future directions of DMT research.

## Materials and methods

The present research was conducted as a systematic review and meta-analysis following the Preferred Reporting Items for Systematic Reviews and Meta-analyses (PRISMA) statement [25].

### Literature search

A systematic literature search was conducted on Web of Science, PubMed, Scopus, and Cochrane Library to retrieve eligible published studies until October 2024 using a constructed search strategy of relevant search strategy (("Multiple sclerosis"OR"MS patients") AND ("vitamin D"OR"cholecalciferol"OR"vitamin D supplementation") AND ("relapse"OR"relapses"OR"annual relapse rate"OR"ARR") AND ("EDSS"OR"Expanded Disability Status Scale") AND ("timed 10-foot walk"OR"T25FW"OR"timed 25-foot tandem walk"OR"TTW10")). Furthermore, the references of the relevant articles and previous reviews were searched for potential additional studies.

### Study selection

Two authors independently screened the titles and abstracts of the retrieved references, and potential studies were screened against the inclusion/exclusion criteria. In case of discrepancies, they were resolved by a third author. We included RCTs, including adult patients with any subtype of MS, that compared an intervention group receiving a high dose of vitamin D to a control group receiving a placebo or low dose of vitamin D, regardless of vitamin D form, and reporting the therapeutic effects on clinical or radiological outcomes such as EDSS, ARR, relapses, and magnetic resonance imaging (MRI) lesions, fatigue, and quality of life (QoL). We included only articles in the English language. Observational studies, reviews, and conference papers were excluded.

### Data extraction and outcome measures

The final included articles underwent data extraction using a pre-specified extraction sheet by two independent authors, and a third author reviewed the final data. Extracted data included methodological and design data, sample size, patient characteristics, age and gender, vitamin D supplement dose and type, outcome measures, and principal conclusions. Primary outcome measures included change in EDSS, change in ARR, number of patients with relapses, and new MRI T2 lesions. Secondary outcome measures included timed 25-foot walk (T25FW), vitamin D levels, fatigue severity scale (FSS) change, and QoL change.

### Quality assessment

To assess the risk of bias, we used the Cochrane risk-of-bias 2 tool (RoB 2), a series of questions that evaluate the trial design, methodology, and reporting [26]. For each study, two authors independently assessed the risk of bias, and a third author resolved any differences. Then, studies were classified as having “high risk”, “some concern”, or “low risk” [26].

### Data synthesis and analysis

A narrative synthesis of the qualitative data was conducted based on the extracted data. Review Manager (RevMan) software (version 5.4) was used for the meta-analysis. Dichotomous outcomes were pooled as odds ratios (OR) with a 95% confidence interval (CI), and continuous outcomes were presented as mean differences (MD) or standardized mean difference (SMD) with a 95% CI. Studies were weighted according to the inverse variance method. A p-value of ≤ 0.05 was deemed statistically significant. The chi-square and I^2^ tests were applied to test for heterogeneity. The data were deemed heterogeneous if the chi-square and p-value were < 0.1 and I2 was above 50%. Two models were used for pooling data depending on the heterogeneity: a random effects model for heterogeneous data and a fixed-effect model for homogeneous data. If heterogeneity was unresolvable, the Cochrane leave-one-out method was applied by excluding one study from the analysis [27]. Subgroup analysis was done according to the duration of vitamin D intake.

## Results

### Literature search

**Fig. 1:**
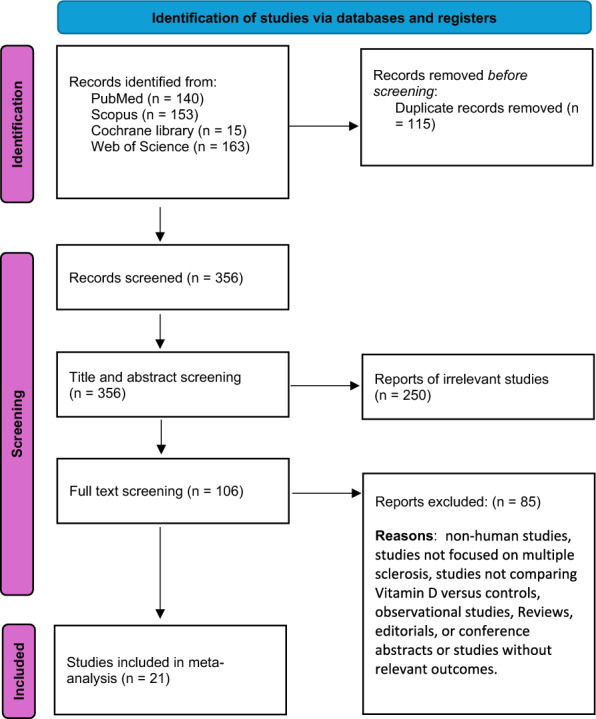
PRISMA flowchart of included studies

The PRISMA flow diagram in Fig. 1 summarizes the search results screening processes and eligibility determinations. Following the search of online databases, 471 studies were retrieved, including 115 duplicates that were subsequently excluded. The titles and abstracts of the remaining 356 articles were screened for eligibility by applying the inclusion and exclusion criteria, which led to the exclusion of 250 studies. The remaining 106 studies were further reviewed in their full texts for eligibility, excluding 85 articles that failed to meet the inclusion criteria. Therefore, 21 studies with 1903 patients were considered eligible for inclusion in this review [18–21, 28–44]. Moreover, the detailed summary and baseline characteristics of the included studies are illustrated in Table 1**.**

**Table 1:** Summary and baseline of the included studies

Study (refs.)	Country	N (V/C)	Age ± SD yrs (V/C)	Male n (V/C)	Vit D form	MS type	Dose (regimen and length)	FU	Key finding
Soilu-Hänninen 2012 [44]	FI	34/32	39 ± 7.8/35 ± 7.3	13/12	D3	RRMS	20 000 IU wk⁻^1^ (0.5 mg)	1 yr	Add-on D3 to IFNB ↓ MRI activity
Smolders 2019 [19]	NL	24/16	37 ± 2.8/40 ± 3.5	7/5	D3	RRMS	7 000 IU d⁻^1^ × 4 wk then 14 000 IU d⁻^1^	1 yr	High-dose D3 ↔ NfL
Stein 2011 [43]	AU	11/12	34 ± 4.9/44.5 ± 1.9	4/3	D2	RRMS	6 000 vs 1 000 IU d⁻^1^	0.5 yr	No added benefit of high-dose D2
Smolders 2011 [42]	NL	174/174	NR	NR	D3	RRMS	7 000 IU d⁻^1^ × 4 wk → 14 000 IU d⁻^1^ × 92 wk	2 yr	Population-level response data
Shaygannejad 2012 [41]	IR	25/25	38.6 ± 8.4/37.9 ± 7.9	3/3	Calcitriol	RRMS	0.5 µg d⁻^1^	1 yr	No effect on EDSS/relapse
Rolf 2017 [40]	NL	20/20	38.5 ± 7.8/37.6 ± 9.6	6/8	D3	RRMS	14 000 IU d⁻^1^	1 yr	↔ depressive symptoms
Cassard 2023 [39]	US	89/83	34.5 ± 7.1/34.2 ± 7.7	28/14	D3	RRMS	600 vs 5 000 IU d⁻^1^	2 yr	High-dose D3 ↔ relapse risk
Hupperts 2019 [20]	NL	113/116	34.1 ± 8.0/33.5 ± 9.3	37/37	D3	RRMS	14 007 IU d⁻^1^	1 yr	No added effect on NEDA-3
Kampman 2012 [38]	NO	35/33	40 ± 7.3/41 ± 6.0	11/9	D3	NR	20 000 IU wk⁻^1^	2 yr	↔ bone loss; no MS benefit
Etemadifar 2015 [37]	IR	6/9	27.7 ± 2.4/30.0 ± 3.9	0/0	D3	NR	50 000 IU wk⁻^1^	0.5 yr	↑25(OH)D, ↓EDSS & relapses in pregnancy
Dörr 2020 [36]	DE	28/25	41 ± 11.1/45 ± 9.0	8/8	D3	RRMS	High: 20 000 IU + 400 IU alt-days vs 400 IU d⁻^1^	1.5 yr	Therapeutic benefit unclear
Camu 2019 [35]	FR	63/66	38.4 ± 9.3/36.7 ± 8.4	13/27	D3	RRMS	100 000 IU wk⁻^1^	2 yr	Possible benefit in low 25(OH)D + IFNβ
Burton 2010 [21]	CA	25/24	41.1 ± 7.4/39.9 ± 8.6	4/5	D3	NR	10 000 IU d⁻^1^ × 12 wk	1 yr	Safe; immunomodulatory signals
Achiron 2014 [34]	IL	80/78	41.3 ± 9.8/40.8 ± 8.7	21/19	Alfacalcidol	NR	1 µg d⁻^1^	0.5 yr	↓ fatigue; ↑ QoL
Holmøy 2017 [33]	NO	35/33	40 ± 7.5/41 ± 5.7	11/9	D3	RRMS	20 000 IU wk⁻^1^	2 yr	↔ bone mass density
Ashtari 2016 [32]	IR	47/47	31.4 ± 7.6/34.6 ± 10.1	NR	D3	RRMS	50 000 IU every 5 d for 3 mo	0.25 yr	↑ mental QoL
H. Steffensen 2011 [31]	NO	35/33	41 ± 5.8/39.7 ± 7.3	NR	D3	NR	20 000 IU wk⁻^1^	2 yr	↔ bone mineral density
Toghianifar 2015 [30]	IR	44/45	31.5 ± 7.6/34.6 ± 10.1	9/5	D3	RRMS	50 000 IU every 5 d × 12 wk	0.25 yr	↓ IL-17 levels
Golan 2013 [29]	IL	24/21	43 ± 12.3/44.7 ± 10.7	5/8	D3	NR	4 370 vs 800 IU d⁻^1^	1 yr	Dose-dependent ↓ IL-17; safe
Mosayebi 2011 [18]	IR	26/33	37 ± 9/35 ± 9	9/8	D3	NR	300 000 IU mo⁻^1^	0.5 yr	↑ TGF-β & IL-10
S. Sotirchos 2016 [28]	US	19/21	41.3 ± 8.1/38.8 ± 8.8	5/7	D3	RRMS	10 400 vs 800 IU d⁻^1^	0.5 yr	Safe; ↓ IL-17; immune modulation

(V/C) = vitamin D/Control; FU = study follow-up duration

25(OH)D, 25-hydroxy-vitamin D; AAR, annual relapse rate; AU, Australia; CA, Canada; DE, Germany; FI, Finland; FR, France; IL, Israel; IR, Iran; NL, Netherlands; NO, Norway; US, United States; D2, ergocalciferol; D3, cholecalciferol; EDSS, Expanded Disability Status Scale; FLS, flu-like symptoms; FU, follow-up; IFNB/IFNβ, interferon beta; IL, interleukin; IU, international unit; MRI, magnetic resonance imaging; MS, multiple sclerosis; RRMS, relapsing–remitting multiple sclerosis; NEDA-3, No Evidence of Disease Activity; NfL, neurofilament light chain; NR, not reported; QoL, quality of life; T25FW, Timed 25-Foot Walk

### Summary of included studies

Twenty-one studies were included in this analysis, encompassing 1903 patients, of whom 381 were male (20.1%) and 1522 were female (79.9%). The mean age was 37.9 years in the vitamin D group and 38.3 years in the Control group. Most of the patient has Relapsing–Remitting Multiple Sclerosis. All the studies were RCTs, as shown in Table 1.

### Quality assessment

**Fig. 2:**
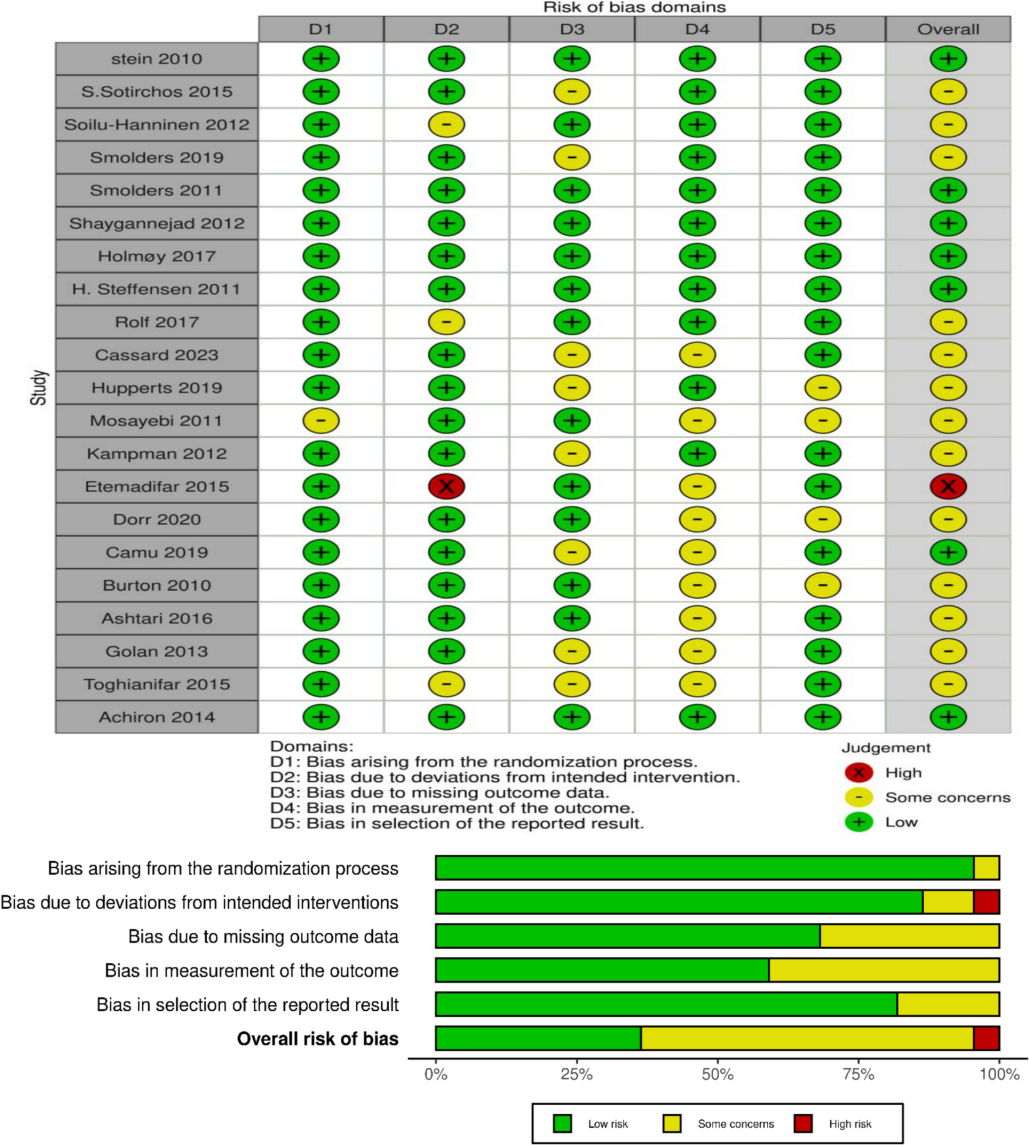
Cochrane risk-of-bias (RoB 2) tool for randomized trials

Based on the RoB 2 tool [26], among the included studies, seven were rated as having a low risk of bias across all domains, indicating a strong methodological approach. However, 13 studies identified some concerns as receiving an unclear rating in at least one domain, while only one study rated a high risk of bias (Fig. 2).

### Outcomes measured

#### Expanded Disability Status Scale (EDSS)

**Fig. 3:**
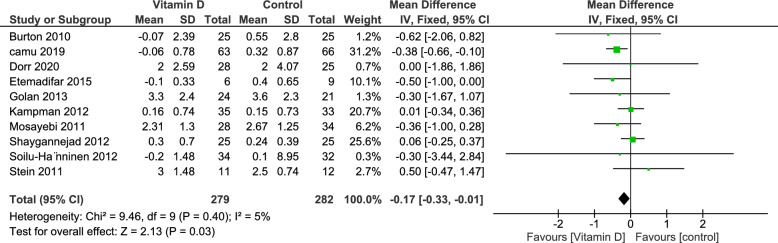
Analysis of Mean difference of EDSS Scores between Vitamin D group and control group

Ten studies reported the EDSS Scale outcome, with 561 patients, of whom 279 were in the vitamin D group and 282 in the Control group. There was a significant decrease in EDSS favoring the vitamin D group (MD = − 0.17; 95% CI [− 0.33, − 0.01], p-value = 0.03). Heterogeneity analysis demonstrated no statistical evidence for heterogeneity (I^2^ = 5%, p-value = 0.40) (Fig. 3).

#### Annual Relapse Rate (ARR)

**Fig. 4:**
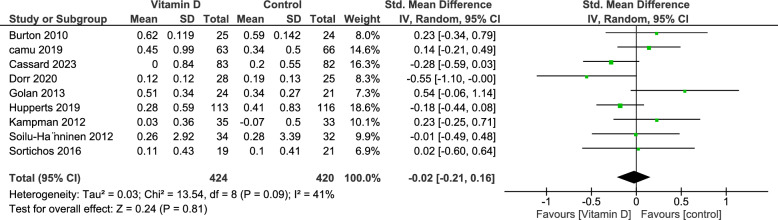
Analysis of Standardized Mean difference of Annual Relapse Rate (ARR) between Vitamin D group and control group

Annual Relapse Rate (ARR) refers to the mean number of relapses per patient per year, calculated by dividing the total number of relapses by the number of patient-years. Nine studies reported the AAR outcome with 844 patients, of which 424 were in the vitamin D group and 420 in the control group. There was no statistically significant difference between both groups (SMD = − 0.02, 95% CI [− 0.21, 0.16], p-value = 0.81). Heterogeneity analysis demonstrated no heterogeneity (I^2^ = 41%, p-value = 0.09) **(**Fig. 4**).**

#### Relapse

**Fig. 5:**
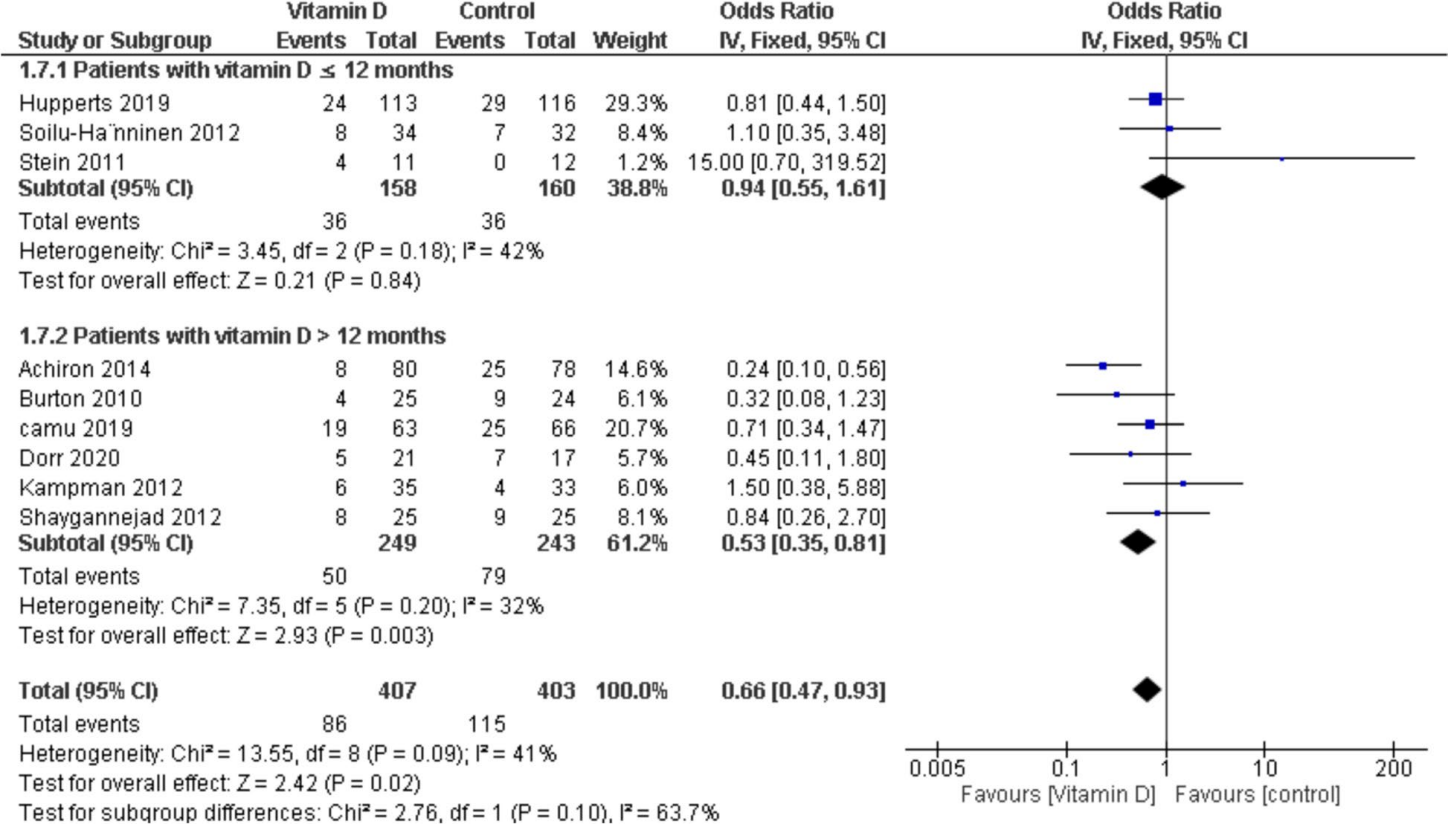
Analysis of Odds Ratio for Patients who experience relapses during research with different administration time of vitamin D

Relapse refers to the binary measure of whether a patient experienced any relapse during the study period (yes/no). Nine studies reported relapse outcome in 810 patients, of which 407 were in the vitamin D group and 403 in the Control group. There was a statistically significant decrease in relapse rates in favour of the vitamin D group (OR = 0.66, 95% CI [0.47, 0.93], p-value = 0.02). There was no heterogeneity among studies (I^2^ = 41%, p-value = 0.09) (Fig. 5).

A subgroup analysis was conducted based on the duration of vitamin D administration, dividing patients into two groups: those receiving vitamin D for ≤ 12 months and those receiving it for > 12 months.

a. For patients who took vitamin D for ≤ 12 months:

The relapse outcome demonstrated no significant change between both groups (OR = 0.94, 95% CI [0.55, 1.61], p = 0.84) with no heterogeneity (I^2^ = 42%, p = 0.18).

b. For patients with > 12 months of vitamin D administration:

There was a statistically significant decrease in relapse rates in favor of the vitamin D group (OR = 0.53, 95% CI [0.35, 0.81], p = 0.003), and no heterogeneity was observed (I^2^ = 32%, p = 0.20).

#### New T2 lesions

**Fig. 6:**
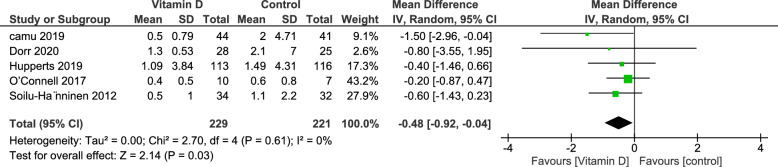
Analysis of Mean difference of New T2 lesions between Vitamin D group and control group

Five Studies reported the New T2 lesion outcome in 450 patients, of which 229 were in the vitamin D group and 221 in the Control group. The data showed a significant decrease in the incidence of new T2 lesions in favor of the vitamin D group (MD = − 0.48, 95% CI [− 0.92, 0.04], p-value = 0.03); there was no heterogeneity (I^2^ = 0%, p-value = 0.61) (Fig. 6).

#### The timed 25-foot walk (T25FW)

**Fig. 7:**
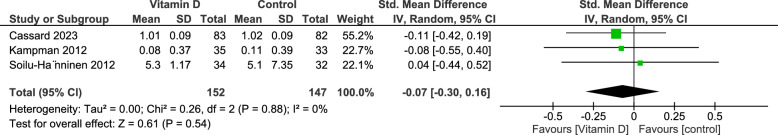
Analysis of Standardized Mean difference of T25FW, timed 10 foot walk between Vitamin D group and control group

Three Studies reported the timed 25-foot walk outcome in 299 patients, of which 152 were in the vitamin D group and 147 in the Control group. There was no statistically significant difference between both groups (SMD = −0.07, 95% CI [− 0.30, 0.16], p-value = 0.54). There was no heterogeneity (I^2^ = 0%, p-value = 0.88) **(**Fig. 7**).**

#### 25-hydroxyvitamin D (25(OH)D)

**Fig. 8:**
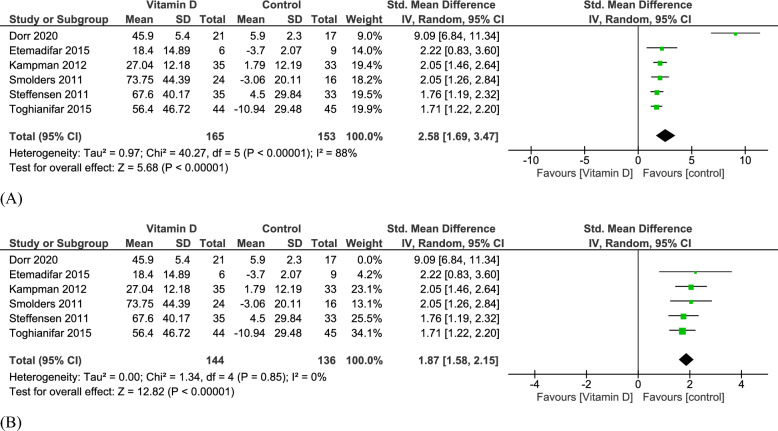
**A** Analysis of Standardized Mean difference of 25(OH)D levels between Vitamin D group and control group. **B** After making Sensitivity analysis by removing One study

Six studies reported 25(OH)D levels in 318 patients, with 165 in the vitamin D group and 153 in the control group. There was a significant increase in the 25(OH)D levels favoring the vitamin D group (SMD = 2.58, 95% CI [1.69, 3.47], p-value < 0.00001). Heterogeneity analysis indicated high heterogeneity (I^2^ = 88%, p-value < 0.00001) **(**Fig. 8A**).** The heterogeneity was resolved upon excluding Dörr 2020, with results showing no significant heterogeneity (I^2^ = 0%, p-value = 0.85), while the overall effect remained unchanged during sensitivity analysis **(**Fig. 8B**).**

#### Fatigue Severity Scale (FSS)

**Fig. 9:**
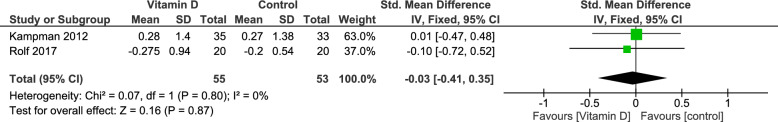
Analysis of Standardized Mean difference of Fatigue Severity Scale (FSS) between Vitamin D group and control group

Two Studies reported the Fatigue Severity Scale outcome in 108 patients, of which 55 were in the vitamin D group and 53 in the Control group. There was no statistically significant difference between both groups (SMD = − 0.03, 95% CI [− 0.41, 0.35], p-value = 0.87). There was no heterogeneity (I^2^ = 0%, p-value = 0.80) (Fig. 9).

#### Quality of life (QoL)

**Fig. 10:**
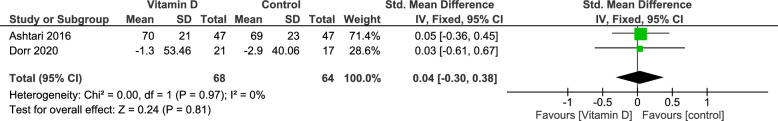
Analysis of Standardized Mean difference of Quality of life (QoL) between Vitamin D group and control group

Two Studies reported the QoL outcome in 132 patients, of which 68 were in the vitamin D group and 64 in the Control group. There was no statistically significant difference between both groups (SMD = 0.04, 95% CI [− 0.30, 0.38], p-value = 0.81). There was no heterogeneity (I^2^ = 0%, p-value = 0.97) **(**Fig. 10**).**

## Discussion

We found that EDSS, relapse rates, and new T2 lesions were significantly lower and favored the vitamin D group. In contrast, 25(OH)D levels were significantly higher and favored the vitamin D group. However, outcomes such as ARR, T25FW, FSS, and QoL showed no significant differences between the vitamin D and control groups. Subgroup analysis indicated a relapse rate reduction only in those treated for more than 12 months, suggesting duration-dependent benefits of vitamin D.

The current study, involving 21 RCTs and 1903 participants, stands out for its larger sample size compared to Mahler et al. (9 RCTs, 867 participants) [24], Yuan et al. (9 RCTs, 718 participants) [7], and James et al. (5 RCTs, 254 participants) [1]. This larger dataset enhances the reliability and robustness of the findings **(**Table 2**).** For instance, the EDSS significantly favored the vitamin D group in the current study. In contrast, Mahler et al. [24] and Yuan et al. [7] reported no significant changes in EDSS scores. However, it is essential to highlight that even though the reduction in EDSS was statistically significant, it may not be clinically relevant, as the observed decrease was only 0.17 points (MD = −0.17; 95% CI [− 0.33, − 0.01], p-value = 0.03). Still, it is crucial to highlight that EDSS itself has several limitations as an outcome measure [45, 46], and some studies reported an unchanged median in the EDSS score for over five years [47]; hence, the significance of results obtained in our meta-analysis requires further investigation—our findings of reduced relapse rates with vitamin D supplementation contrast with some previous studies and meta-analyses. We believe several factors may explain this discrepancy. First, our meta-analysis included more recent trials and a larger sample size (21 studies with 1,903 patients) than earlier analyses. For instance, the relapse rates in our study showed a significant reduction favoring the vitamin D group. In contrast, James et al. [1] and Yuan et al. [7] reported no significant changes in relapse rates. A critical insight from our analysis is the duration-dependent effect of vitamin D supplementation. Subgroup analysis revealed that only patients receiving vitamin D for > 12 months demonstrated a significant reduction in relapse rates (OR = 0.53, p = 0.003), whereas those treated for ≤ 12 months showed no benefit (OR = 0.94, p = 0.84). This temporal threshold likely accounts for why earlier studies with shorter follow-ups failed to observe a protective effect. Vitamin D's immunomodulatory actions may require prolonged exposure to manifest clinical benefits, particularly in conditions like multiple sclerosis, where DMT often necessitates long-term adherence.

**Table 2:** Comparison of the current study with the previous meta-analyses

Outcome	Current study	Mahler et al. 2024 [24]	Yuan et al. 2021 [7]	James et al. 2013 [1]
No. of RCTs	21	9	9	5
No. of total participants	1903	867	718	254
EDSS	MD = − 0.17; 95% CI [− 0.33, − 0.01], p-value = 0.03	MD = − 0.02; 95% CI [− 0.37, 0.41], p-value = 0.91	MD = − 0.02; 95% CI [− 0.35, 0.31], p-value = 0.91	
	(I^2^ = 5%, p-value = 0.40)	(I^2^ = 0%, p-value = 0.56)	(I^2^ = 54%, p-value = 0.05)	
ARR	SMD = − 0.02; 95% CI [− 0.21, 0.16], p-value = 0.81	MD = − 0.03; 95% CI [− 0.08, 0.02], p-value = 0.26		
	(I^2^ = 41%, p-value = 0.09)	(I^2^ = 38%, p-value = 0.13)		
Relapse	OR = 0.66; 95% CI [0.47, 0.93], p-value = 0.02		OR = 0.85; 95% CI [0.59, 1.32], p-value = 0.40	OR = 0.98; 95% CI [0.44, 2.17], p-value = 0.96
	(I^2^ = 41%, p-value = 0.09)		(I^2^ = 8%, p-value = 0.36)	(I^2^ = 36%, p-value = 0.18)
New T2 Lesions	MD = − 0.48; 95% CI [− 0.92, 0.04], p-value = 0.03	MD = − 0.59; 95% CI [− 1.24, 0.07], p-value = 0.08		
	(I^2^ = 0%, p-value = 0.61)	(I^2^ = 30%, p-value = 0.23)		
T25FW	SMD = − 0.07; 95% CI [− 0.30, 0.16], p-value = 0.54			
	(I^2^ = 0%, p-value = 0.88)			
25(OH)D	SMD = 2.58; 95% CI [1.69, 3.47], p-value < 0.00001			
	(I^2^ = 88%, p-value < 0.00001)			
FSS	SMD = − 0.03; 95% CI [− 0.41, 0.35], p-value = 0.87			
	(I^2^ = 0%, p-value = 0.80)			
QoL	SMD = 0.04; 95% CI [− 0.30, 0.38], p-value = 0.81			
	(I^2^ = 0%, p-value = 0.97)			

EDSS, expanded disability status scale; AAR, annual relapse rate; T25FW, timed 10-foot walk; FSS, fatigue severity scale; QoL, quality of life

Moreover, comparable insignificant results were found in reporting on ARR [24] with the previous meta-analyses [1, 7, 24]. Other meta-analyses did not report the results of 25(OH)D, FSS, T25FW, and QoL. Despite the significance we observed in the increasing levels of 25(OH)D, it is already known that vitamin D supplements may increase 25(OH)D levels [48]. However, this increase in 25(OH)D levels might not directly indicate a correction in the clinical condition of the patients; as reported earlier, vitamin D deficiency causes, but it might be essential to prevent vitamin D deficiency causes neurological and psychiatric disorders, such as MS, Parkinson's disease, Alzheimer's disease [49]. Also, it could be a common risk factor by interfering with modulating the exacerbation of hereditary brain disorders and/or debilitating the recovery from brain stressors through adulthood [50]. Notably, despite the complex origin of these diseases and the involvement of genetic and environmental factors, the mechanism of how polymorphisms in both the vitamin D receptor (VDR) gene [51] and vitamin D metabolism genes [52] leave several unanswered questions. For instance, regarding the relapse, Ramagopalan et al. [53] have identified a vitamin D response element (VDRE) in the promoter region of HLA-DRB1*15 and *16 haplotypes. However, it may not be exclusive to these haplotypes or HLA-DR1 [54]. Also, vitamin D was strongly linked to reduced contrast-enhancing lesions in HLA-DRB115:01-negative individuals. In HLA-DRB115:01-positive individuals, higher vitamin D levels were associated with a lower relapse risk for those with one allele copy but not those with two copies [47].

Regarding QoL, several studies reported improvement in QoL after vitamin D supplementation in patients with MS [55, 56]. However, only two trials were included in our analysis, and their pooling analysis reported significant results, but we should be cautious with these findings. First, in the Dörr et al. (2020) study [36], the sample size was small (21 patients administered vitamin D versus 17 controls). In the Ashtari et al. (2016) [32], the unadjusted model had no significant differences. However, in the adjusted model based on sex, age, disease duration, and EDSS scores, the vitamin D group significantly differed in mental health composite with the placebo group, 62.41 ± 13.99 vs. 60.99 ± 17.99 (p-value = 0.041).

Regarding FSS, while the pooling analysis of the two studies included showed insignificant results, a dedicated meta-analysis on the influence of vitamin D supplementation on fatigue in patients with MS by López-Muñoz et al. [57] showed a significant reduction in fatigue was perceived when vitamin D supplementation was compared with a control group: MD =  − 0.18 (95% CI − 0.36 to − 0.01). The authors included all studies that reported fatigue regardless of the scale used, such as the Modified Fatigue Impact Scale and Fatigue Impact Scale. Hence, reporting only the FSS scale in our analyzed studies was the main limitation and required cautious interpretation of the results. Therefore, our observations and analysis suggest that the effect of vitamin D—even if it exists—may not be consistent across all individuals and requires further investigation.

An interesting finding in our study was concerning new T2 lesions, as we found a significant reduction in new T2 lesions favoring the vitamin D group. In contrast, Mahler et al. showed a non-significant trend towards fewer new T2 lesions. While Mahler et al.'s results did not reach statistical significance, the direction of the effect aligns with the current study. Moreover, the study by Mowry et al. [47] reported that each 10 ng/mL higher vitamin D level was associated with a 15% lower risk of developing new T2 lesions later. These results and our analysis align with translational studies showing that vitamin D_3_ inhibits myelin basic protein-specific T-cells, increases CD4 + CD25 + T-regulatory cells, and decreases microglia and astrocyte activation [58, 59]. Therefore, we recommend studies with more participants to confirm these findings.

### Clinical implications

In our pooled analysis, vitamin D supplementation produced a statistically significant –0.17-point shift on the Expanded Disability Status Scale, yet this falls well below the 0.5- to 1.0-point change usually considered a clinically meaningful slowing of disability in MS [60]. Although the odds of experiencing any relapse dropped by roughly one-third, that advantage appeared only in trials lasting longer than 12 months and was not seen in the large VIDAMS randomized trial, which found no difference in ARR despite 5,000 IU/day of cholecalciferol [39]. MRI data showed a slight reduction in new T2 lesions, which aligns with the high-dose D-Lay MS study (100,000 IU every two weeks) in clinically isolated syndrome and early relapsing disease, implying that both dose and stage may modulate benefit [61]. Supplementation reliably raised serum 25(OH)D and is linked experimentally to dampened pro-inflammatory cytokine production and enhanced regulatory-T-cell activity, mechanisms that give biological plausibility to the modest clinical effects [62, 63]. Hence, correcting vitamin D deficiency is necessary and may confer incremental benefits, particularly with long-term use or in early disease. However, the absolute effects on disability, relapse risk, and MRI activity are modest, so vitamin D should be viewed as a low-risk adjunct rather than an evidence-established disease-modifying therapy until larger, dose-stratified trials powered for clinically meaningful endpoints are completed.

### Strengths and limitations

Our study's larger number of participants provided greater statistical power, allowing us to identify differences in EDSS and confirm the trend in new T2 lesions previously observed by Mahler et al. (2024) [24]. Additionally, we reported several new outcomes not mentioned before, such as T25FW, 25(OH)D, FSS, and QoL. Our study has several important limitations that must be acknowledged. First, the analysis of FSS and QoL outcomes was restricted to only two studies, which limits the robustness of these findings. Additionally, our analysis reported the QoL outcome as an adjusted value.

In contrast, unadjusted models from other studies, such as those referenced in our discussion, may have shown significant improvements. Furthermore, the FSS scale was the sole fatigue measure included in our analysis, despite other studies using alternative scales (e.g., Modified Fatigue Impact Scale) demonstrating significant associations with vitamin D supplementation. These methodological differences may explain discrepancies between our results and prior research results.

Another critical limitation is the potential for confounding due to variations in DMT among study groups. Since vitamin D supplementation is an adjuvant therapy and not the primary treatment for MS, differences in baseline DMT regimens or adherence could introduce bias, affecting the interpretation of outcomes. This heterogeneity in concomitant therapies may explain the contradictory findings regarding QoL and FSS compared to other studies reporting beneficial effects of vitamin D.

Finally, while we observed high heterogeneity in 25(OH)D levels (I^2^ = 88%) across studies, this was resolved without altering the overall results. Notably, this heterogeneity in 25(OH)D levels could stem from variability in baseline deficiencies and assay discrepancies across studies. Similarly, non-standardized MRI protocols (e.g., field strength, lesion quantification) and latitudinal differences (e.g., deficient high latitude vs. equatorial cohorts) risk biased outcomes. These limitations highlight the need for future research with larger, more homogeneous cohorts, standardized outcome measures, and rigorous DMT variability control to better clarify vitamin D's role in MS management. Also, stratification of populations by baseline vitamin D status is highly recommended.

## Conclusion

Vitamin D supplementation improved EDSS, relapse rates when administered over 12 months, new T2 lesions, and 25(OH)D levels. However, it is essential to note that the improvement in EDSS, although statistically observed, may not be clinically meaningful, raising questions about the practical implications of these changes for patients with MS. Additionally, no significant associations were found between vitamin D supplementation and improvements in ARR or T25FW. Our findings on FSS and QoL also present complexities. At the same time, their results appeared comparable between the vitamin D-supplemented and the control groups; this should not be interpreted as an insignificant association with fatigue or QoL. Instead, it reflects the limitations of the FSS scale and the unadjusted QoL values from the studies included in our analysis, warranting cautious interpretation. While signs of potential benefit exist, the evidence remains mixed, highlighting the need for future research. Larger, well-designed, long-term studies with comprehensive and appropriately adjusted analyses are required to clarify the role of vitamin D in MS management.

## Data Availability

No datasets were generated or analysed during the current study.
